# Work Availability and Exergy Analysis

**DOI:** 10.3390/e20080597

**Published:** 2018-08-10

**Authors:** Pouria Ahmadi, Behnaz Rezaie

**Affiliations:** 1 Institute for Sustainability, Energy, and Environment (ISEE), University of Illinois at Urbana-Champaign (UIUC), Urbana, IL 61802, USA; 2 Applied Energy Research Laboratory, Department of Mechanical Engineering, University of Idaho, 875 Perimeter Drive, MS 0902, Moscow, ID 83844-0902, USA

**Keywords:** Exergy, availability, efficiency, optimization, exergy destruction

Exergy analysis, which is based on the second law of thermodynamics, is a potential tool to identify the sources, magnitude and location of the irreversibility in energy systems. Exergy analysis can assist researchers, engineers and students for system design, analysis, assessment, optimization, and performance evaluation of various energy systems. In this Special Issue, we have tried to attract researchers to submit their interesting and novel research works related to work availability, exergy and exergo-economic analyses. 18 high quality papers after several rounds of reviews were accepted and published in this Special Issue. A brief overview and summary of the individual contributions are given in the following:

The first contribution was by Hooshmand et al. [1] who investigated the numerical study of the magnetic field effects on the heat transfer and entropy generation aspects of a power law fluid. The study deals with the investigated entropy production in a steady state fully developed forced convection incompressible flow over an axisymmetric stretching sheet such that the stretching wall was subjected to different wall temperatures and the Rosseland approximation model. They also studied the effects of several parameters on entropy generation. Thermal radiation coefficient, Prantdl number, Hartmann number, and Brinkmann number were the parameters considered and the results were explained. Lake et al. [2] studied the use of exergy analysis to quantify the effect of lithium bromide concentration in an absorption chiller. They used EES software for the simulation purpose and tried to numerically determine the optimal LiBr concentration when the exergy destruction rate was minimized. The results showed that the absorber, the generator and the condenser were the largest contributions to the exergy destruction rate at 30%, 31% and 28%, respectively. The results also showed that an increase in concentration was shown to decrease the maximum cooling capacity by 3% and increase the exergy destruction of the generator by 4.9%.

Pérez-García et al. [3] studied the second law analysis of a mobile air conditioning system with internal heat exchanger using low global warming potential (GWP) refrigerants. In their study, they considered R152a, R1234yf and R1234ze as low GWP refrigerants and establishing R134a as baseline. They also considered the effect of Sub-Cooling and superheating effects on the cases. The results showed that the most efficient operation of the system resulted when using the R1234ze refrigerant. The exergy loss distribution and the heat transfer capability in the subcritical Organic Rankine Cycle (ORC) was carried out by He et al. [4]. The ORC system considered in this study consists of a pump, an evaporator, an expander and a condenser. The net power output was selected as the objective function and the optimal value of the exergy loss distribution of the subcritical Organic Rankine Cycle system by using R245fa was determined. They also performed a parametric study to see the influences of heat source temperature, the evaporator pinch point temperature difference, the expander isentropic efficiency and the cooling water temperature rise on the exergy loss distribution of subcritical ORC system are comprehensively discussed. It was found that there exists a critical value of expander isentropic efficiency and cooling water temperature rise, respectively, under certain conditions. 

Sciubba and Zullo [5] published a work titled Exergy Dynamics of Systems in Thermal or Concentration Non-Equilibrium. The paper approached the problem of the existence and quantification of the exergy of non-equilibrium systems. The results showed that the non-equilibrium exergy was always larger than its equilibrium counterpart and constitutes the “real” total exergy content of the system, i.e., the real maximum work extractable from the initial system. Multi-Objective Optimization for Solid Amine CO_2_ Removal Assembly in Manned Spacecraft was carried out by Pang et al. [6]. The purpose of this paper was to determine the optimal design parameters for the solid amine Carbon Dioxide Removal Assembly. The optimization variables consist of adsorption cycle time, solid amine loading mass, adsorption bed length, power consumption and system entropy production. They applied a NSGAII optimization based on genetic algorithm to determine the optimal design parameters. Demirkaya et al. [7] studied the Thermal and Exergetic Analysis of the Goswami Cycle Integrated with Mid-Grade Heat Sources. They studied a combined Power and Cooling Cycle that employs an Ammonia–Water mixture. The cycle combines a Rankine and an absorption refrigeration system. They investigated the waste heat energy with temperature range between 35 and 100 °C and its effect on system performance.

An Improved System for utilizing low-temperature waste heat of flue gas from coal-fired power plants was carried out by Huang et al. [8]. In this research article, an improved system to efficiently utilize the low-temperature waste heat from the flue gas of coal-fired power plants was proposed based on heat cascade theory. They conducted an in-depth analysis of the energy-saving characteristics of the improved waste heat utilization system (WHUS) and the conventional WHUS. Mugica et al. [9] investigated the exergy analysis of a parallel-plate active magnetic regenerator with nanofluids. They analyzed the energetic and exergy performance of an active magnetic regenerative refrigerator using water-based Al_2_O_3_ nanofluids as heat transfer fluids. A 1D numerical model has been extensively used to quantify the exergy performance of the system. Fontalvo et al. [10] studied the energy, exergy and economic evaluation comparison of small-scale single and dual pressure organic Rankine cycle integrated with low-grade heat sources. The results illustrated that the maximum power output was lower than 45 KW while the temperature of the heat source varies in the range 100–200 °C.

Energy and exergy assessment of a pilot parabolic solar dish-Stirling system was carried out by Gholamalizadeh and Chung [11]. The location of the solar system was a sunny desert region in Iran (Kerman). They showed energy losses occurred in the receiver and in the Stirling engine, while the major exergy losses occurred in the receiver and in the concentrator. The maximum collector energy efficiency and total energy efficiency were reported as 54% and 12.2%, respectively, in July, while from November to February the efficiencies were very low. Similarly, the maximum collector exergy efficiency and total exergy efficiency were reported as 41.5% and 13.2%, respectively, in July. 

Dorosz et al. studied possible liquefied natural gas (LNG) exergy recovery systems for transportation since in most LNG-fueled vehicles, exergy of LNG was destroyed during the regasification process [12]. Their approach was “cold energy” recovery systems as the enthalpy of LNG, to use for cooling power in air conditioning or refrigeration by four systems. The single-stage and two-stage direct expansion systems, an ORC system, and a combined system (ORC + direct expansion) were the four exergy recovery systems that applied LNG as a low temperature heat sink to generate electric power. Authors analyzed and optimized exergy of these LNG power cycles. They reported exergetic efficiencies in a range of 20–36%, parallel with a network for a range of 214–380 kJ/kgLNG.

Huang et al. compared the integration of three retrofit concepts for waste heat recovery via ORC, in-depth boiler–turbine integration, and coupling of both for maximizing the system-level heat [13]. Their comparison was in terms of thermodynamic and economic performances. They reported that the in-depth boiler-turbine integration provided a better temperature match of heat flows involved for different fluids and multi-stage air preheating, which showed a major upgrading of power output (23.99 MW) in comparison with the original system (6.49 MW). The limitation of the ultra-low temperature (75–135 °C) heat available from the flue gas for ORC was the reason for this improvement which was mostly contributed by the reduction of exergy destruction within the boiler subsystem. R11 as the ORC working fluids leads to maximizing the power output because of the low-grade heat source. The most economic completive solution with a payback period of only 0.78 year was the in-depth boiler–turbine integration. The ORC option with payback time of 2.26 years was the least attractive for a sole application. Authors concluded that, by integration of both concepts, a net power output of 26.51 MW and a payback time of almost one year were the best option.

Spanghero et al. applied the first and second laws of thermodynamics to the human body to estimate the quality of the energy conversion during muscle activity [14]. Authors believed that deficiency in the literature made difficulties in evaluating energy and exergy in some activities. Therefore, they used work as an input in the exergy model to evaluate two types of exercises: weight lifting and aerobic exercise on a stationary bicycle. They conducted a study of the aerobic and anaerobic reactions in the muscle cells for predicting the metabolic efficiency and muscle efficiency during exercises. Oxygen consumption, carbon dioxide production, skin and internal temperatures, and performed power were the physiological data that were measured. They concluded that the exergy efficiency was 4% in the weight lifting, while it could increase to 30% for aerobic exercises.

Jing et al. investigated building cooling based on the exergo-economics through solar refrigeration, i.e., solar/natural gas-driven absorption chiller (SNGDAC), solar vapor compression–absorption integrated refrigeration system with parallel configuration (SVCAIRSPC), and solar absorption-subcooled compression hybrid cooling system (SASCHCS) [15]. Authors considered three types of buildings for cooling: Type 1, the single-story building; Type 2, the two-story and three-story buildings; and Type 3, the multi-story buildings. They also considered two Chinese cities, Guangzhou and Turpan. They concluded that SNGDAC was considered as a suitable solution for Type 1 buildings in Turpan, since natural gas consumption was negligible and resulted in the lowest product cost flow rate. SVCAIRSPC was more suitable for Type 2 buildings in Turpan since there was a higher actual cooling capacity of absorption subsystem with a lower fuel and product cost flow rate. Finally, SASCHCS was the most cost-effective, considering that exergy destruction and product cost flow rate were both the lowest when applied for all types of buildings in Guangzhou and Type 3 buildings in Turpan.

Since ORC is known as a promising technique to exploit waste heat from Internal Combustion Engines (ICEs), Liu et al. investigated the waste heat recovery systems that could be designed based on engine rated working conditions [16]. Authors modeled an off-design Medium Cycle/Organic Rankine Cycle (MC/ORC) system by interconnecting the component models that allowed the behavior estimation of system off-design. Methodology was the sliding pressure control that applied to balance the variation of system parameters, while operational variable was evaporating pressure. The results indicated that, by the reduction of engine load, the MC/ORC system effectively recovered waste heat, while the maximum net power output, thermal efficiency and exergy efficiency linearly declined. Finally, by the drop of engine load, the gas–oil exchanger and turbine increase exergy loss increased, while the evaporator and condenser exergy loss decreased. 

The investigation on the impact of muscle and fat percentages on the exergy behavior of the human body in different environmental conditions was conducted by Garcia et al. [17]. The focus of the study was to relate the thermal comfort indicators with exergy rates, resulting in a second law perspective. They modeled human body with four layers: core, muscle, fat and skin with some simplifications. Author validated the model based on a set of environmental conditions and body compositions. They concluded that the area normalization (Watts per square meter) could be applied to the exergy transfer to environment, while the destroyed exergy itself was adequate to estimate the thermal sensation when the model was exposed to environmental temperatures lower than that considered for the thermal neutrality condition. To calculate the thermal comfort for environments with higher temperatures than that calculated for thermal neutrality, the destroyed exergy and the rate of exergy transferred to the environment must be considered in calculations.

Castro et al. [18] investigated the impact of temperature and air velocity on the exergy of the continuous-convection drying of onion. The energy and exergy for the onion drying chamber were derived to monitor the exergy efficiency, exergy loss rate, exergetic improvement potential rate, and sustainability index. Authors reported that the boost in temperature and air velocity had direct impact on exergy loss rates increase since the overall heat transfer coefficient varies with these operation conditions. In addition, a boost of air velocity caused the exergy efficiency to increase due to the energy utilization for the moisture evaporation. Meanwhile, an increase of temperature caused the exergy efficiency to decrease due to the free moisture being diffused from the internal structure to the surface. Thus, to condense environmental impact of the process, the parameters must be adjusted for an optimum exergy efficiency of the process.
